# Counterfactual quantum key distribution with untrusted detectors

**DOI:** 10.1016/j.heliyon.2023.e13719

**Published:** 2023-02-13

**Authors:** Ya-Qian Lin, Meng Wang, Xiu-Qing Yang, Hong-Wei Liu

**Affiliations:** a College of Science, Inner Mongolia University of Technology, Hohhot 010051, China; b China Information Technology Security Evaluation Center, Beijing 100085, China

**Keywords:** 0000, 1111, Quantum key distribution, Security proof, Counterfactual communication

## Abstract

Compared with the traditional BB84 protocol, the counterfactual quantum key distribution (QKD) does not rely on any signal travelling in the quantum channel, and therefore can present a security advantage where Eve cannot fully access signal. However, the practical system may be damaged in a scenario where the devices are untrusted. In this paper, we analyze the security of counterfactual QKD in untrusted detectors scenario. We show that the requirement to disclose “which detector clicked” has become the main loophole in all counterfactual QKD versions. An eavesdropping scheme which is similar to the memory attack on device-independent QKD could break its security by exploiting detectors' imperfections. We consider two different counterfactual QKD protocols and analyze their security against this major loophole. One is a modified Noh09 protocol, which would be secure in untrusted detectors context. Another is a variant of counterfactual QKD with high efficiency (Phys. Rev. A 104 (2021) 022424) against a series of detectors side-channel attacks as well as against other attacks that exploit detectors imperfections.

## 1 Introduction

Quantum key distribution (QKD) [1] is a promising application to distribute secret bits based on quantum mechanics. The wider application of QKD focus on transmission distance and secure distribution service. Recently, the optimized twin-field QKD system has overcome the barriers of 140 dB channel loss and 837.8 km fibre distance has been tested [2]. Meanwhile, measurement-device-independent QKD has matured to real-world deployment over fibre network, including a network upgrade based on existing phase-encoding QKD system [3] and a robust and adaptable network [4]. Since the first protocol was proposed by Bennett and Brassard in 1984 [1], quantum communication systems generally rely on the transmission of signals to transfer information. However, Noh [5] proposed counterfactual quantum key distribution (CQKD) in 2009 to enable Alice and Bob share a secret key without any qubit travelling between them. The CQKD can be implemented with multiphoton signals because Eve cannot determine the photon number without having access to the mode in Alice's site. Since then many researchers followed his work, which make it becoming a promising application of quantum. The security of CQKD protocol has been proved using an ideal single photon source [6] and weak coherent state [7] by assuming any type of Trojan-horse attack can be detected by the two communication parties. In 2012, the experimental CQKD with 1 km fiber distance has been achieved [8]. In 2013, a counterfactual quantum secure direct communication protocol [9] was proposed based on interaction-free measurement and the quantum Zeno effect [10], [11], [12] to achieve 100% efficiency of information transmission. The principle-and-proof experiment was implemented in Ref. [13]. Following the concept, the counterfactual Trojan-horse attack was proposed by Ref. [14], in which the eavesdropper may steal the secret information by “ghost” photons and can spontaneously avoid being detected. In 2021, in Ref. [15], Rao et al. proposed a novel CQKD to improve the efficiency by including noncounterfactual key bits. In Rao21 protocol, the key generation rate reaches 25%, which is the level of BB84 protocol.

However, most QKD system is normally insecure because the devices do not typically conform to the theoretical modes considered in the security proof. The security of CQKD with untrusted devices could easily be compromised. For example, imperfect detectors in Bob's site would make CQKD vulnerable to an invisible photon and a delay-photon Trojan-horse attack [16]. Furthermore, the proposed attack in Ref. [17] showed that CQKD is vulnerable to the detector blinding and an experimental implementations should include explicit countermeasures against it. In particular, in Ref. [18], the authors showed that the original CQKD having the requirement to disclose “which detector clicked” in public channel to allow Alice and Bob to know which bit was accepted for a key was compromised in a device-independent (DI) context. The authors then proposed a DI CQKD based on single-photon entanglement protocol. In this paper, we consider the counterfactual QKD in untrusted detectors scenario. We show that the requirement to disclose “which detector clicked” has become the main loophole in all the CQKD system. An eavesdropping scheme which is similar to the memory attack on DI QKD [19], [20], [21], [22] is applicable to the CQKD system. We then consider two different CQKD protocols and subsequently proper CQKD, which would be secure in untrusted detector context. In either case, the key idea is to allow Alice or Bob to encode his bit information by using the spatial degree freedom of the incoming photons. In one case, we propose a modified Noh09 protocol that relaxes the requirement to disclose “which detector clicked” while still allowing the legitimate parties to share a key. In another case, we propose a variant of Rao21 protocol to close “which detector clicked” and analyze its security against a series of side-channel attacks. We have more security assumptions in untrusted detectors scenario than the device-independent (DI) QKD. Compared with the DI framework having low noise tolerance, however, it is more feasible to make a proof-of-principle demonstration.

## 2 Security analysis

**Figure 1:**
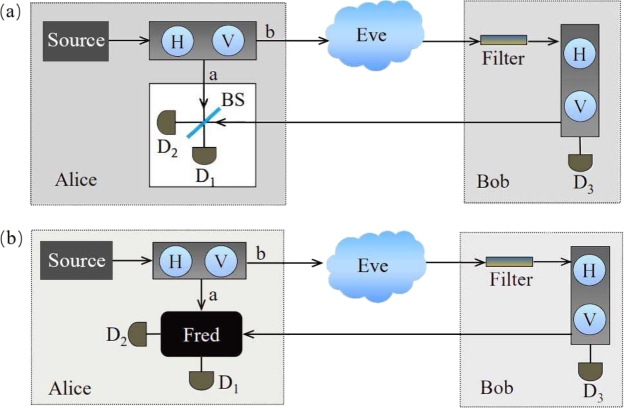
(a) Original CQKD scheme. Alice splits the encoded signal into two paths *a* and *b*. Only the click at D_1_ provides Alice with a conclusive knowledge of Bob's bit without any signal travelling in the quantum channel. (b) Untrusted detector device variant. Alice's detector device is unknown quantum device, called Fred. Security is compromised since Fred and Eve can together steal information.

In the original CQKD protocol, as shown in Fig. 1(a), Alice encodes a random polarization, chosen from {0,π}, on the single-photon and splits the encoded signal into two paths *a* and *b*. The resulting state is an entangled state of single photon and vacuum that is accessible to both Alice and Bob, as defined by Eq. (1) [5]

(1) $${{{|\Phi \rangle =\frac{1}{\sqrt{2}}{(|1\rangle }_{b}|0\rangle }_{a}-|0\rangle }_{b}|1\rangle }_{a}).$$

Only the pulse in path *b* can travel to Bob's site and will be reflected back if Alice and Bob choose different bits. In Alice's site, he implements a single-photon interference with a Michelson-type interferometer. The interference effect will cause the photon to be detected at D_2_ with certainty. However, if Alice and Bob choose the same bit, the pulse will be blocked and the interference is destroyed. In this case, detector D_1_ and D_2_ click with probability 25% respectively, and detector D_3_ clicks with probability 50%. At the end of transmission, only the click at D_1_ provides Alice with a conclusive knowledge of Bob's bit. In this configuration, all the imperfections in practical QKD system need to be overlooked.

However, practical implementation of CQKD unavoidably contain imperfections which has led to various side-channel attacks. In a DI context, the authors analysed the security of QKD. They proposed a set of black box, for Alice and Bob, which could completely simulate the expected performance of CQKD while allowing Eve to knowing the key bits. They considered two different strategies to demonstrate how the black boxes should work by distributing tripartite states. In one scheme, an entangled bipartite state of Alice and Bob is separable from a third state. Suppose that the state being distributed is given by Eq. (2) [18]

(2) $${{{{{|\Phi \rangle }_{m}=\frac{1}{\sqrt{2}}{(|1\rangle }_{b}|0\rangle }_{a}-|0\rangle }_{b}|1\rangle }_{a})⊗|m\rangle }_{b},$$

where *m* ∈{H,V} denotes Alice's choice of polarization, either with horizontally or vertically polarization. Since the state ${|m\rangle }_{b}$ can be measured perfectly without disturbing the entangled state, Eve would know with certainty which bit is accepted as a key base on the revelation of “which detector clicked”. In another scheme, the tripartite state are completely separable. One way of closing this loophole is to close “which detector clicked”. However, Alice and Bob could not share a key without announcing “which detector clicked” in the original CQKD. Hence, the authors proposed a CQKD in DI context. Assure Alice and Bob share a maximally entangles states, it is possible for them to extract a secure key without information on “which detector clicked”. Following these results, they propose a new equivalent CQKD based on single-photon entanglement [23], [24] in DI framework. A secure key can be extracted when Alice and Bob observe a violation of the Clauser-Horne inequality [25]. Device-independent QKD is based on a test for a violation of a Bell inequality, even if the users' devices are not fully characterize. However, existing DI QKD security proofs have low noise tolerances, making practical implementation challenging.

Since most identified security loopholes in conventional QKD are associated with detectors, we consider the CQKD in untrusted scenario. We show that the revelation of “which detector clicked” could give Eve access to the information despite the state being a maximally entangled. As shown in Fig. 1(b), Alice's detector device is an unknown device, called Fred, which could be manufactured by the adversary Eve. Eve and Fred can together access the qubit in Alice's site. It is assumed that Fred can not communicate the keys with Eve outside Alice's site, because otherwise no secure key can be established.

Here, we show that Qi's attack [26] that exploits detectors' imperfections of the receivers for detector-device-independent (DDI) is applicable to CQKD system. The DDI QKD protocol claimed to be immune to detector side-channel attacks. However, Qi presented that combined with Trojan-horse attack, a simple scheme would allow the Bell-state measurement to leak key information to the outside through the interval of the BSM results. Following this method, we describe the attack on CQKD in detail. Suppose that Fred successfully detects the signal sent by Alice by detector D_1_ which is used for a key. Meanwhile Fred also knows Alice's bit as she can perform a measurement to distinguish the two polarization states perfectly. Then Fred encodes Alice's random bit on the time delay *r* between adjacent case of detector D_1_ clicking. If Alice's bit value is 1 (0), Fred will output a result only when *r* is an even (odd) number. Alice has to mention when D_1_ clicks for key generation purpose and allows Eve to know which bit is going to be accepted as a key. When Alice announces all the events at D_1_, Eve can decode Fred's information through the time interval of D_1_ clicking. Fred can perfectly simulate the protocol by carefully controlling the reporting rate to match it with the QKD system. This attack is similar to the memory attack on DI QKD. Although the security of DI QKD has been proved by evaluating collective attack, the overwhelming memory attack can still break the DI QKD system.

Although Qi's attack could be used in DDI QKD as well as in CQKD, the implementation conditions are totally different in the two scenarios. In DDI QKD, Eve could learn the whole secret key based on the assumption that she identifies Bob's bit using her own photon. Each time Eve requires sending her own optical state along with signal state into Bob's system to encode Bob's bit. Fred will then use the result of this measurement, combined with the BSM on legitimate qubit, to give Eve information mutual with the key. The key for a successful eavesdropping is whether Eve can successfully send her photon into Alice's site. It is clear that if Bob has a filter to prevent Eve's photon, Fred could not send information to the outside. However, in the CQKD Fred knows precisely well on Alice's bit without the implementation of the Trojan-horse attack, therefore no level of sophistication in the protocol can prevent her from learning the key. Compared to the DDI QKD, it is easier to implement the above attack in CQKD system.

## 3 Modified CQKD

### 3.1 A modification of Noh09 protocol

**Figure 2:**
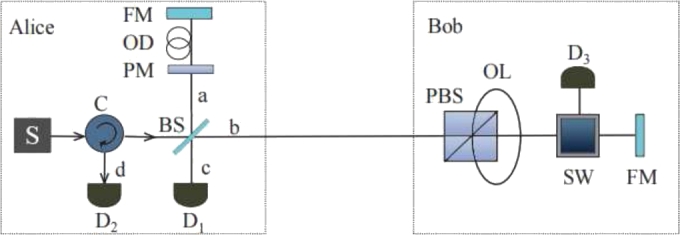
Modified CQKD scheme. Alice introduces a random phase, chosen from 0 or *π* on the pulse in path *a*. Either detection by D_1_ or detection by D_2_ could allow Alice share a key with Bob. Thus Alice and Bob relax the requirement to disclose “which detector clicked”. In our model, the detectors in Alice's site do not need to be trusted.

When Alice's detectors are untrusted, we propose a modified CQKD protocol to close the main loophole. We assume Alice's state preparation is characterized and trusted. In particular, the beam splitter (BS) can be regarded as a trusted transmitter. However, the detectors in Alice's site do not need to be trusted. In our scheme, Alice encodes his bit information by using the spatial degree of freedom of the pulse in path *a*. This is done with a phase modulator that applies a random phase, chosen from 0 or *π*, to each pulse *a*. Our proposed setup is shown in Fig. 2. Alice triggers the photon source (S) and prepare a single-photon state in either horizontally polarised state |H〉 as bit 0 or vertically polarised state |V〉 as bit 1. The single-photon state passing a 50:50 BS is given by Eq. (3)

(3) $${{{|\Psi \rangle =\frac{1}{\sqrt{2}}{(|P\rangle }_{b}|0\rangle }_{a}+{\left(-1\right)}^{s}|0\rangle }_{b}|P\rangle }_{a}),$$

where P∈{H,V} represent the horizontally and vertically polarize states, ${|0\rangle }_{i}$ denotes the vacuum state with i∈{a,b} and s∈{0,1} designates the phase of the pulse 0 and *π* in path *a*, respectively. Then the pulse in path *b* enters the input port of the polarising beam splitter (PBS) on Bob's site. The state |H〉 will be transmitted, while |V〉 will be reflected and travel through optical loop (OL). Bob will choose between horizontally and vertically polarize states to represent his bit using switch (SW). Based on Alice's choice of phase, we will consider the following two cases. When Alice and Bob have different bits, the pulse in path *b* will be reflected by Faraday mirror (FM) and the pulses in the two modes are recombined at BS. With the creation operators $a^{†}$ and $b^{†}$ of this pulse in two paths, the state transformation at the BS can be described by Eq. (4)

(4) $${\left(-1\right)}^{s}a^{†}|0\rangle ,b^{†}|0\rangle ,$$

respectively.

After the interference, the quantum state at the two detectors becomes one of Eq. (5)

(5) $$[1-{\left(-1\right)}^{s}]c^{†}|0\rangle ,[1+{\left(-1\right)}^{s}]d^{†}|0\rangle ,$$

where $c^{†}$ and $d^{†}$ are the creation operators at the two detectors, respectively. This means if s=0, the interference will cause a click at detector D_2_. While if s=1, there is a click at detector D_1_. When Alice and Bob have the same bit, the pulse in path *b* will be detected by detector D_3_. In the event that Alice's state collapses to ${{|0\rangle }_{a}|P\rangle }_{b}$, a click will happen at D_3_. On the other hand, Alice's state will collapse to ${{|P\rangle }_{a}|0\rangle }_{b}$, and therefore can obtain a click at D_1_ or D_2_ with equal probability. In the case where Alice's phase is 0, only a detection by D_1_ provides Alice with a conclusive knowledge of Bob's bit. On the contrary, with the phase *π*, only the click at D_2_ allows Alice to have complete knowledge of the key. Alice does not require to announce the information on “which detector clicked” when a key is generated. Since a click of either D_1_ or D_2_ implies that Alice could share a key with Bob, Eve could not have known which bit is being accepted as a key. As a result, by not revealing information on “which detector clicked”, a secure key can be extracted in the untrusted scenario.

### 3.2 A variant of Rao21 protocol

Different from Noh09 protocol, the basic idea of Rao21 protocol is to increase the key generation rate by including noncounterfactual bits. In Noh09 protocol, the key is generated when the interference in the interferometer is destroyed. In the absence of interference, the probability of Bob's detector (D_3_) being triggered is 50%, and the probability of Alice's two detectors being triggered is 25%. The key can only be generated when Alice's specific detector (D_1_) is triggered. In Rao21 protocol, however, the key is generated when D_3_ is triggered, and the case of triggering D_1_ is only used for security checks, thereby improving the key generation rate. The authors also pointed out that there is noiseless attack in the improved scheme, but it can be resolved by Alice and Bob's independent flip operation on the reflected particles without reducing the key generation rate.

Despite having different encoding rules, Bob's operation in Rao21 protocol follows the same procedure as Noh09 protocol, i.e., reflecting the photon of polarization |H〉 while blocking |V〉, or reflecting |V〉 while blocking |H〉. The CQKD with high efficiency still has a feature that Eve has access to a signal traveling over both Bob-Alice channel and Alice-Bob channel, and therefore the quantum channel itself is a potentially open door for an eavesdropper into Bob's apparatus. For these CQKD the eavesdropping schemes involve side-channel attacks, as well as other attacks that exploiting detectors' imperfections [16], [27]. As a result, the Trojan-horse attacks on the original CQKD system [16] is still valid when the detectors are untrusted. In the first strategy, Eve sends a fake photon with a delay time, which is unlikely to be detected because this photon is outside Bob's detection time window. In the second strategy, Eve sends an invisible photon that is far from the wavelength range that Bob's detector works. Eve need to determine only whether her photon is blocked by Bob or not. If Eve capture her photon, Eve's and Bob's bit values are different; otherwise, their two bit values are equal. Thus Eve can Obtain Bob's bit information without being detected.

The previous Trojan-horse attack [16] can be performed on badly designed systems using current technology. Normally, it has been proven that secure QKD is nevertheless possible, provided that any other detection should be eliminated by properly designed filter and any encoding device should be activated only during a time as short as possible. However, even these two countermeasures cannot completely prevent the Trojan-horse attack. In particular, when Bob's detectors are untrusted, the above memory attack can allow Fred to leak information to the channel through the interval of Bob's triggering event. The timing of different polarized photons passing through the SW is different. Suppose that Fred obtains a detection at a time window corresponding to returning |H〉 photon while blocking |V〉 photon, he will output a result through an even time interval. Otherwise, he will output a result through an odd time interval.

**Figure 3:**
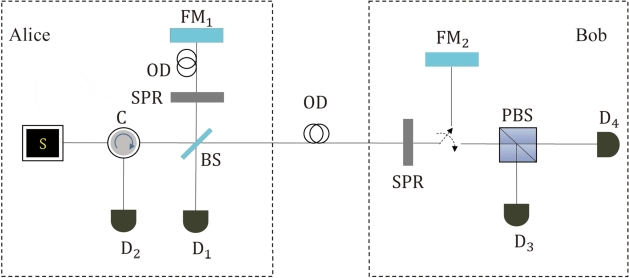
A variant of Rao21 protocol. Using a SW Bob randomly chooses to reflect the incoming pulse, or measure its polarization. If Bob obtains a detection at D_3_ or D_4_, these noncounterfactual bits will be used as key bits. If Bob's detection action does not detect a photon, Alice's detection at D_1_ will forms counterfactual bits. The noncounterfactual bits are indicated to be secure by counterfactual bits. In our model, the detector in Bob's site is unknown quantum device.

We make a slightly simplified Rao21 protocol to effectively close major security loophole. See Fig. 3, our protocol works as follows: (1) Alice prepares a single-photon in the polarization state |H〉 or |V〉 to a Michelson-type interferometer. One of the arms of interferometer remains in her site, while the other stretches out to Bob's station. (2) Alice and Bob independently perform the flip operation on a fraction of instances using a switching polarization rotator (SPR). In contrast to the Rao21 protocol, Bob applies the flip operation on the incoming particle. Bob encodes a random bit $s_{1}$ on her flip operation followed by measurement. If he flips the polarization of the photon to its orthogonal state, his bit is 1; otherwise his bit is 0. (3) With a SW Bob either reflects the incoming pulse, or measures its polarization. In the case of reflection, if Bob's flip action matches Alice's action, then the reflected photon will be detected deterministically at D_2_. (4) When Bob detects the pulse, the key bit is generated at D_3_ or D_4_, whereas the security check ia accomplished at D_1_. Bob determines the value $s_{2}$ and his bit is 0 (1) corresponding to the polarization state |H〉 (|V〉). (5) Since Bob use two different degrees of freedom of the incoming photons, Bob announces his detection and records $s_{B}=s_{1}⊕s_{2}$ as his sifted key.

Note that our setup in Bob's station is different from the one in former protocol. One is Bob conducts measurement by applying a SW prior to PBS and any photon in a certain polarization state will be absorbed. Just like the one-way configuration, we make it impossible for a photon to travel back when a key is generated and reduce the maximal information that an adversary can gain using Trojan-horse attack. In other words, Eve's Trojan-horse attack is quite limited since it avoids the problem of sending Trojan-horse photon and monitoring the reflected signal from Bob in key generation case. Consider Eve's eavesdropping is limited to the Alice-Bob channel when a key is generated, this does reduce the security analysis of the corresponding one-way CQKD, compared to those former CQKD systems. Since Eve cannot capture any photon to probe Bob's laboratory when a key is generated, it is naturally secure against the invisible photon and delay-photon Trojan-horse attack. As far as the key generation is concerned, however, the procedure is equivalent to polarization-dependent reflecting or absorbing operation on each photon in Rao21 protocol. Another is Bob applies the SPR to the incoming pulse. If Bob reflects the pulse, Bob's flip operation in our modification works equally well in Rao's protocol when the noiseless attack is taken into account. If Bob detects the pulse, his operation is considered as a way of coding to improve the security, making it immune to the memory attack. Security comes from the fact that Bob's encoding encloses the flip operation $s_{1}$ which is regarded as a trusted transmitter. Even if Fred obtains a detection at D_3_ or D_4_ and encodes $s_{2}$ on an even or odd time interval, Eve only learns the detected polarization value $s_{2}$ and therefore knows little about Alice's key bits. As a result, the modified version can remove their major security loophole from CQKD implementations.

Next, we demonstrate that the modified scheme is robust against other attacks that exploits the imperfections of Bob's detectors. Note that the detector blinding attack described in Ref. [17] is based on the fact that Eve could produce a strong blinding signal and monitor the signal coming back from Bob. In our scheme, however, there is no possibility for a photon to travel back when a key is generated. We will assume that Bob's receiver has only one active detector, while the other detector is disabled. For example, Eve shines bright light onto Bob's detector D_3_ to make it enter linear-mode operation. In this mode, the detector is no longer sensitive to single-photon pulses, except for strong light. Once D_3_ is blinded, Bob measures Alice's signal and only obtains a click at D_4_. However, the click at one detector only corresponds to the value $s_{2}$ and could not disclose any information by sending light signals. We consider as well the time-shift attack that allow Eve to know “which detector clicked” by exploiting the detection efficiency mismatch between Alice's two detectors [28], [29]. In this type of attack, Eve shifts the arrival time of each signal sent to Bob such that only one detector can produce a click each given time. In our scenario, however, Eve cannot determine whether or not an active detector corresponds to Alice's bit when Bob's encoding encloses the polarization flip. Specifically, Eve makes D_3_ produce a click at a certain time. Alice's bit may be 0 or 1 depending on Bob's flip operation. As a result, our scheme is still secure against attacks by controlling the efficiency of detectors.

## 4 Conclusion and discussion

We show that the revelation of “which detector clicked” has become the main loophole in all the CQKD versions. We introduce an eavesdropping strategy that breaks the security of DDI QKD by exploiting detectors' imperfections on the CQKD system. Our attack is valid provided the legitimate parties have to disclose “which detector clicked” to establish the key strings in the CQKD, even when they share the perfect source. We propose two modified CQKD protocols to close this major loophole. This requires that Alice and Bob use two different degrees of freedom of the single photons to encode their bit information. One is a modification of Noh09 protocol, in which Alice could share a key with Bob by introducing a random phase. Another is a variant of Rao21 protocol, in which Bob applies a SPR on the incoming particle against detectors side-channel attacks as well as against other attacks that exploit detectors imperfections.

A security proof of Rao21 protocol based on the most general attack is still missing, although its advantage over Noh09 protocol is outstanding. Note that in Ref. [30], the author showed that the CQKD is insecure through a high lossy channel. The security comes from the reason that the error rate is as high as that under Eve's attack when the loss rate exceeds 50%. The security of CQKD seems to be untrusted detectors, it is assumed to be protected from well characterized state preparation process. A security proof based on collective attack and security analysis concerning practical source and channel loss needs further research.

## CRediT authorship contribution statement

Ya-Qian Lin, Meng Wang: Conceived and designed the analysis; Analyzed and interpreted the data; Wrote the paper.

Xiu-Qing Yang, Hong-Wei Liu: Contributed analysis tools or data; Wrote the paper.

## Declaration of Competing Interest

The authors declare no conflict of interest.

## Data Availability

Data will be made available on request.
